# P-39. Trends in Pertussis Antibody Titers after DTaP Vaccination of Healthcare Workers at a Tertiary Children's Hospital in Japan

**DOI:** 10.1093/ofid/ofae631.246

**Published:** 2025-01-29

**Authors:** Mihoko Furuichi, Tomohiro Katsuta, Yukitsugu Nakamura, Eisuke Suganuma, Naoki Shimizu

**Affiliations:** Saitama prefectural Children's Medical Center, Saitama-shi, Saitama, Japan; St. Marianna University School of Medicine, Kawasaki, Kanagawa, Japan; St Marianna University School of Medicine, Kawasaki, Kanagawa, Japan; Saitama prefectural Children's Medical Center, Saitama-shi, Saitama, Japan; St. Marianna University School of Medicine, Kawasaki, Kanagawa, Japan

## Abstract

**Background:**

In Japan, diphtheria-tetanus-acellular pertussis (DTaP) is used for adults instead of tetanus toxoid, reduced diphtheria toxoid, and acellular pertussis (Tdap) vaccine. DTaP vaccinations for healthcare workers in obstetrics and pediatrics have been recommended since 2019 in Japan. However, data on the prevalence rate of pertussis antibodies among health care workers are limited. This study aims to evaluate pertussis antibody titers before and after DTaP vaccination to establish an optimal vaccination schedule for healthcare workers.

**Methods:**

This study was a prospective observational study targeting staff members who received DTaP vaccination at our hospital after 2019. We investigated age, gender, history of pertussis, and history of vaccination with pertussis-containing vaccines at the time of participating in this study. Anti-PT-IgG antibodies (EIA method) were measured once a year in the remaining serum from staff health examinations. We defined 40 EU/mL as the antibody-positive threshold.

**Results:**

There were 1454 subjects, with a median (IQR) age of 33 (26-44) years and 349 (24%) were male. Twenty-one (1.4%) had a history of pertussis, and the reason for diagnosis was antibody testing in 8, symptoms in 7, and unknown in 6. Thirty-one (2%) received a pertussis-containing vaccine within 10 years at the time of participating in this study. Antibody positivity rates were 16% (194/1222), 42% (383/928), 35.3% (226/639), and 25% (105/416) before, one year, two years, and three years after final DTaP vaccination, respectively. Within 1028 seronegative subjects before vaccination, 25% seroconverted after vaccination; during 108 subjects of which who could follow their titer continuously, 48% maintained the antibodies for up to three years. In one case, the person did not seroconvert but developed antibodies up to 100 IU/mL two years after vaccination. During the study period, no pertussis cases were diagnosed, and no outbreaks were observed in our hospital.

**Conclusion:**

In this study, 84% of Japanese healthcare workers did not have sufficient pertussis antibodies before DTaP vaccination. Following a single dose of DTaP vaccination, 25% showed positive pertussis antibody titers, of which 45% maintained effective antibody levels for 3 years.

**Disclosures:**

**All Authors**: No reported disclosures

